# Concept of a Novel Glass Ionomer Restorative Material with Improved Mechanical Properties

**DOI:** 10.3390/jfb14110534

**Published:** 2023-10-24

**Authors:** Philipp Messer-Hannemann, Henrik Böttcher, Sven Henning, Falk Schwendicke, Susanne Effenberger

**Affiliations:** 1 DMG Dental-Material Gesellschaft mbH, 22547 Hamburg, Germany; 2 Fraunhofer Institute for Microstructure of Materials and Systems IMWS, 06120 Halle (Saale), Germany; 3 Department of Oral Diagnostics, Digital Health and Health Services Research, Charité-Universitätsmedizin Berlin, 14197 Berlin, Germany

**Keywords:** glass ionomer, dental restorative material, novel material concept, particle reinforcement, transmission electron microscopy (TEM), flexural strength, fracture toughness

## Abstract

The objective of this study was to transfer the concept of ductile particle reinforcement to restorative dentistry and to introduce an innovative glass ionomer material that is based on the dispersion of PEG-PU micelles. It was hypothesized that reinforcing a conventional glass ionomer in this way increases the flexural strength and fracture toughness of the material. Flexural strength and fracture toughness tests were performed with the novel reinforced and a control glass ionomer material (DMG, Hamburg, Germany) to investigate the influence of the dispersed micelles on the mechanical performance. Transmission electron microscopy was used to identify the dispersed micelles. Fracture toughness and flexural strength were measured in a 3-point-bending setup using a universal testing machine. Before performing both tests, the specimens were stored in water at 37 °C for 23 h. The fracture toughness (MPa∙m^0.5^) of the novel glass ionomer material (median: 0.92, IQR: 0.89–0.94) was significantly higher than that of the control material (0.77, 0.75–0.86, *p* = 0.0078). Significant differences were also found in the flexural strength (MPa) between the reinforced (49.7, 45.2–57.8) and control material (41.8, 40.6–43.5, *p* = 0.0011). Reinforcing a conventional glass ionomer with PEG-PU micelles improved the mechanical properties and may expand clinical applicability of this material class in restorative dentistry.

## 1. Introduction

Direct restorative dental materials are widely used to replace dental hard tissue that has been lost due to caries, fracture, tooth abrasion, or developmental disorders. To counteract tooth decay, there are many possible material options for direct restorative therapy, e.g., dental amalgam, resin composites, compomers, glass ionomer cements (GICs), and resin-modified glass ionomer cements (RMGICs). The decisive factors for the choice of these materials are very versatile. Whereas resin composites are predestined as a permanent solution to withstand high mechanical loads in occlusally stressed areas or for restorations with high aesthetic demands, GICs are particularly used as a biocompatible, moisture-tolerant, and cost-effective solution in pediatric and geriatric dentistry, where there is generally a high caries risk and a reasonable treatment effort is required due to the low adherence of these patient groups [1,2]. Since caries is the most common disease nowadays, affecting millions of deciduous teeth and billions of permanent teeth [3], the caries-preventing properties of conventional GICs are advantageous for a growing number of patients with special needs compared to other available restorative materials due to their ion-release capabilities, which are assumed to support the remineralization of carious tooth structures. Given the burden of untreated dental caries on the health system and the need to expand primary and preventive dental care, the World Health Organization (WHO) recently included GIC in the list of essential medicines to meet the minimum requirements of a basic health system [4].

GICs are based on the dissolution of a finely powdered reactive fluoro-alumino-silicate glass by an aqueous polyacrylic acid solution that initiates an acid–base reaction of the material [5]. During this chemical setting process, ionic bonds are formed between the carboxyl functional groups of the GIC and the calcium ions in the tooth surface to ensure sufficient adhesion to the dental hard tissue without the use of an intermediate material, thus making the application of adhesive systems unnecessary [6]. In addition to this and the ability of the material to capture and release fluoride ions that can induce remineralization processes when incorporated in the surrounding tooth structure, conventional GICs are considered bioactive direct restorative materials [7,8]. Newer formulations of these restorative materials, referred to as high viscosity GICs, additionally have a higher molecular weight of polyacrylic acid and an optimized size distribution of the incorporated glass fillers, making them less susceptible to water intake compared to older generations [9,10].

Despite the many advantages of these materials and their long history of clinical use, conventional GICs have one significant limitation in terms of their mechanical properties. Low resistance to crack propagation when exposed to high occlusal forces limits their general use as a direct restorative material in load-bearing and extended cavities [11,12,13]. To broaden clinical applicability and benefit from the proven advantages of conventional GICs over other restorative materials, it is essential to improve their mechanical properties without compromising biocompatibility and simplicity of application during the restorative treatment [14]. The clinical potential of this group of materials is demonstrated by higher retention rates of GICs compared to resin composites for cervical lesions in follow-up periods up to five years [15].

Early attempts to increase the flexural strength and resistance to fracture of conventional GICs involved the addition of resin monomers and an associated initiator system to the liquid component of the material. It has been shown that the presence of a polymerized resin component toughens the restorative material and improves the ability to withstand loading in flexure compared to conventional GICs [16]. However, due to the competition of two different setting processes, the resulting material has a complicated structure, and mixture of the setting reactions may jeopardize the reliability of the set material [17]. The light-activated polymerization reaction can also limit the ion exchange within the material and with the surrounding tissue [8]. This might be associated with a limited caries preventive effect and impaired adhesion to the tooth structure [18,19]. RMGICs have the same clinical applications as conventional GICs, though they are not suitable for bulk application and atraumatic restorative treatment (ART) due to the light activation required.

Other approaches for improving the mechanical properties of conventional GICs included metal particle reinforcement with metal additives incorporated into the glass powder, but these have not provided sufficient metal–matrix interfacial bonding, and there was no clear improvement in the strength of these materials compared to conventional GICs [20,21]. The incorporation of reactive and non-reactive glass fibers into conventional glass ionomer formulations, on the other hand, resulted in improved mechanics without compromising the advantages of a biocompatible restorative material [22,23]. Reinforcement with nanoparticles of titanium dioxide or zirconia can reduce the porosity of conventional GICs, which reduces the formation of cracks and leads to an increase in compressive strength [24]. Yttria-stabilized zirconia nanoparticles synthesized by a sol-gel method also showed suitable properties to be potentially used as biocompatible nanofillers for GICs [25]. Altogether, reinforcement of a conventional GIC without the addition of resin monomers is possible but has so far only been confirmed at an experimental stage in a laboratory setting without commercialization and translation into clinical materials [26].

The novel material concept introduced in this study is based on PEG-PU nanoparticles that will form micelles with an amphiphilic elastic structure when dispersed in water. Incorporation of ductile particles inside a brittle matrix is a widely used concept in materials science to increase the fracture resistance and to impede crack initiation and propagation due to energy dispersion caused by plastic deformation of the incorporated particles or the formation of bridging zones inside a propagating crack [27,28,29].

The aim of this study was to transfer the concept of ductile particle reinforcement to restorative dentistry and to introduce a novel material concept that is based on a conventional GIC with dispersed PEG-PU micelles to prevent crack propagation and to improve the mechanical performance of the restorative material without compromising the biocompatibility. It was hypothesized that reinforcing a conventional GIC using this concept increases the flexural strength and fracture toughness of the material, demonstrating its potential for expanding clinical applicability.

## 2. Materials and Methods

**Figure 1:**
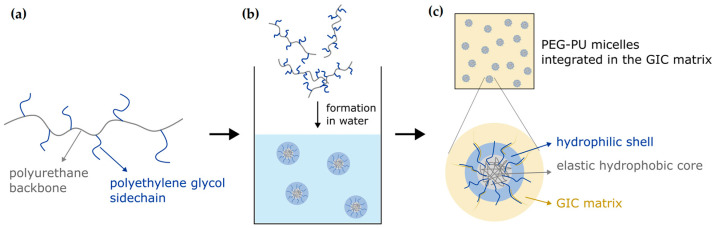
Concept of reinforcing a conventional GIC: PEG-PU nanoparticles (**a**) were dispersed in water to form micelles that are composed of a hydrophobic core and a hydrophilic shell (**b**). Formation of interpolymer complexes with the polyacrylic acid enables firm integration into the GIC matrix (**c**).

The reference material used as control for this study was a highly viscous conventional GIC that was intended to be classified as a restorative material [5]. For the mechanical tests of this study, both reinforced and control, GICs were hand-mixed with a defined powder–liquid ratio of 4.9:1.0 according to the instructions for use provided by the manufacturer (DMG, Hamburg, Germany). The powder consisted of fluoro-alumino-silicate glass and solid polyacrylic acid and the liquid of polyacrylic acid, tartaric acid, and water (DMG, Hamburg, Germany). Reinforcement of the reference material was performed by the integration of ductile particles inside the liquid component of the GIC that were formed in aqueous solution by self-aggregating spherical amphiphilic graft polymers that have a nanosized micelle structure. The graft polymers were composed of a hydrophobic and partially elastic polyurethane (PU) backbone and hydrophilic polyethylene glycol (PEG) side chains. Therefore, the elastic core of a micelle consists of hydrophobic PU and the shell of PEG. Integration into the final cement matrix is potentially ensured due to the interfacial bonding of the hydrophilic and polar PEG surface with the also polar polyacrylic acid in the cement matrix, thereby forming micelle–polyacrylic acid complexes (Figure 1). Consequently, the micelles are assumed to be strongly integrated, unable to freely move through the material.

A range of analytical methods were employed to characterize the control and reinforced GIC: (1) optical verification of the PEG-PU micelles after dispersion in distilled water was performed using transmission electron microscopy (TEM); and to investigate the influence of the dispersed micelles on the mechanical performance of the GIC, (2) flexural strength and (3) fracture toughness tests were carried out as a comparison between the reinforced and the (unaltered) control glass ionomer material without micelles.

### 2.1. TEM

To achieve adequate image contrast and to reduce sensitivity to electron beam damage, a positive staining and fixation technique was applied prior to TEM investigations of the dispersed micelles. An aqueous solution containing deionized water (Reagent Grade A.C.S.) and 2% osmium tetroxide (OsO_4_) with a purity of 99.95% (Science Services GmbH, Munich, Germany) was used for the staining protocol. One drop of this solution was added to 2 mL of a suspension that consisted of distilled water and the dispersed micelles with a concentration of 2 wt.%. After 24 h, the stained suspension was further diluted by the addition of distilled water at a ratio of 1:10. One drop of the stained and diluted suspension was then placed on a carbon-coated TEM grid and dried at room temperature. TEM investigations were performed by means of a FEI Tecnai G2 instrument (FEI Company, Hillsboro, OR, USA) operated at 200 kV in the bright field mode. As a control, a suspension without dispersed micelles was analyzed with the same preparation protocol.

### 2.2. Fracture Toughness

The fracture toughness was measured using 3-point-bending with single-edge-notched specimens (n = 8) according to the ASTM standard E399 [30,31]. After mixing the material, beam-shaped specimens with a width of 4.0 mm ± 0.2 mm, a thickness of 3.0 mm ± 0.2 mm, and a length of 25.0 mm ± 0.5 mm were prepared by application of the mixed GIC into a Teflon mold with the desired dimensions. The mold filled with the cured GIC material was sealed and stored in a humidity chamber at 37 °C for 1 h. After 1 h, the specimens were demolded and stored in water for 23 h ± 1 h at 37 °C. Before carrying out the fracture toughness test, the samples were ground smooth on both sides involved in the load transfer and a 0.8 mm deep notch was sawn into the side of the specimen facing away from the applied force using a diamond cutting disc. A universal testing machine (Zwick Z010, Zwick/Roell, Ulm, Germany) with a loading speed of 0.8 mm/min was used to measure the maximum force *F_max_* before specimen failure occurred. To calculate the fracture toughness *K_1,C_*, the following equation was used [31]:

(1) $$K_{1,C}=\frac{F_{max}\cdot \;S\cdot \;10^{-6}}{B\cdot \;W^{1.5}}\cdot \;f(\frac{a}{W})$$

where *W* is the width of the specimen, *B* is the thickness of the specimen, *S* = 20 mm is the space between the two load supports, and *a* is the depth of the notch. Except the space between the supports, all parameters were measured for each specimen to be able to neglect geometric influences. A detailed description of the function $f(\frac{a}{W})$ is found in the ASTM standard E399 [31].

### 2.3. Flexural Strength

The flexural strength of the investigated materials was measured according to ISO 9917-2:2017 in a 3-point-bending setup using 25 mm × 2 mm × 2 mm specimens (n = 8) [32]. Similar to the test setup that was used to measure the fracture toughness, specimens were stored for 1 h at 37 °C in a humidity chamber inside a Teflon mold. Prior to testing, the specimens were demolded and stored in water for 23 h ± 1 h at 37 °C. Loading of the specimen was done with a speed of 0.8 mm/min until fracture occurred using the Zwick Z010 testing machine. The maximum load *F_max_* at fracture of the specimen was used to calculate the flexural strength *σ* using the following equation [32]:

(2) $$\sigma =\frac{3\;\cdot F_{max}\;\cdot l}{2\;\cdot b\;\cdot h^{2}}$$

with *l* = 20 mm as space between the supports.

### 2.4. Statistics

Statistical analysis was performed using GraphPad PRISM (Version 9, Graphpad Software, San Diego, CA, USA). Due to the low sample number (*n* = 8), the results of the mechanical testing (flexural strength, fracture toughness) were analyzed for statistically significant differences using the non-parametric Mann–Whitney U test. A Type I error level of 0.05 was used for all tests of significance.

## 3. Results

**Figure 2:**
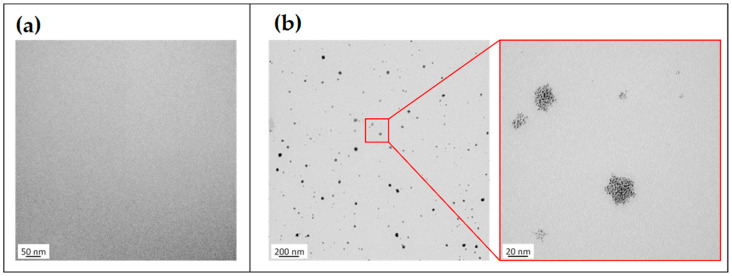
TEM images of the diluted suspensions (1:10) after staining with osmium tetroxide (OsO_4_). (**a**) Control without micelles; (**b**) with micelles.

The formation of osmium-tetroxide-stained micelles after dispersion in distilled water could be verified with the TEM images (Figure 2b). Dissolution of the amphiphilic PEG-PU nanoparticles led to the aggregation of spherical micelles that were homogeneously distributed inside the suspension and averaged 15 nm (10–50 nm). In comparison, the TEM images of the control sample did not show any dispersed particles inside the matrix (Figure 2a), confirming that the visible aggregates inside the modified suspension are the micelles. Occasional agglomerates of multiple micelles might have been formed due to the evaporation process of the liquid during TEM imaging, contributing to the slight variations in the size of the micelles.

**Figure 3:**
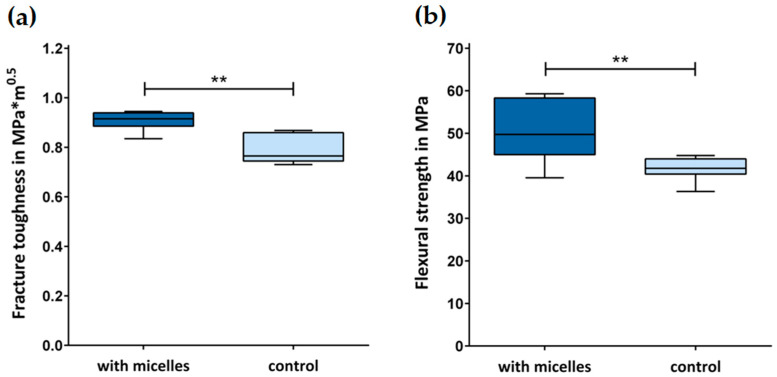
Fracture toughness (**a**) and flexural strength (**b**) of the investigated GICs. The fracture toughness and flexural strength of the material with dispersed micelles were significantly higher compared to the control material (‘**’: *p* ≤ 0.01). The error bars represent the minimum and maximum values of the measured data.

The fracture toughness of the GIC with the dispersed micelles was significantly higher than the control material without micelles (*p* = 0.0078, Figure 3a). An analysis of the fracture surfaces indicated a brittle fracture pattern in both test groups without any differences being visually recognizable. Significant differences were also found in the flexural strength (MPa) between the reinforced and control material (*p* = 0.0011, Figure 3b).

## 4. Discussion

Direct restoration of carious lesions requires dental materials that are durable, biocompatible, and easy to place. For a long period, amalgam was considered the optimal solution for this purpose [33]. However, as amalgam is gradually being phased out due to mercury pollution regulations [34,35], there is a high demand for alternative cost-effective materials with a high simplicity of application to ensure satisfactory affordable treatment for patients and to avoid tooth extraction as the only other treatment option. In addition to resin-based composites as highly aesthetic and load-bearing restorative solution, which, on the other hand, have limitations in terms of biocompatibility, moisture tolerance, and use in high-risk patients [36,37], the reinforcement of conventional GICs is a promising attempt to fill the gap that will be created by further restricting the use of amalgam. Especially when used as a dental sealing material or within an ART approach involving minimal removal of tooth structures with hand instruments, the ion-release capabilities of conventional GICs can reduce the incidence of secondary caries to a much higher extent compared to resin composites or amalgam. To benefit from these advantages and to overcome early restoration failures, the mechanical performance of these materials must be improved in order to maximize the longevity of the restoration. The potential of this group of materials is also perceived by clinicians and manufacturers, leading to a growing interest in research and increased market prospects for modern high-viscosity GIC formulations [14,38].

In this study, PEG-PU micelles were dispersed within the cement matrix to improve the limited mechanical properties of conventional GICs and to increase their potential in dental restorative therapy. Due to their hydrophilicity, the PEG side chains enable homogeneous dispersion and thereby sterically stabilize the micelles to not form agglomerates when distributed inside the liquid component of the GIC. With the micelles integrated into the cement matrix, flexural strength and fracture toughness were investigated as a comparison between a control and the reinforced direct restorative material. With a significant improvement in flexural strength and fracture toughness, our hypothesis that reinforcing a GIC with the dispersion of ductile particles increases mechanical strength can be confirmed. Given the identical base material, the differences in fracture toughness and flexural strength can be directly attributed to the micelles incorporated into the GIC matrix. Fracture toughness and flexural strength are considered important laboratory parameters for the survival rate of direct restorative materials and correlate moderately with relevant clinical outcome parameters [39]. While fracture toughness is associated with the resistance of materials to crack propagation, flexural strength is an indicator of the resistance to deformation and fracture initiation. Thus, both properties are relevant indicators for long-term stable direct restorations. The values reported in the literature for flexural strength and fracture toughness of conventional GICs vary widely, mainly due to a wide range of different test conditions and specimen preparations. This makes it extremely challenging to draw any reliable conclusions when comparing the results of this study to values found in the literature. However, the results of this study seem superior compared to other conventional GICs [16,40,41,42]. Using a similar test protocol, Moberg et al. reported flexural strength values of 18 to 34 MPa for commercial conventional GICs after 24 h storage in a physiologic phosphate-buffered saline solution at 37 °C [16]. François et al. stored their specimens in water for two weeks at 37 °C before performing the flexural strength testing in a three-point bending setup. For the conventional GIC in their material selection, a flexural strength of 22.7 ± 6.9 MPa was measured [40]. This is in the same range as measurements of Faridi et al. [41]. They measured a mean flexural strength of 23.1 MPa after storage of conventional GIC specimens in artificial saliva for 24 h at 37 °C. They also found a peak flexural strength after two weeks of storage in artificial saliva, while the samples showed reduced flexural strength after four weeks. Battula et al. tested flexural strength and fracture toughness of a conventional GIC with a test protocol similar to this study and measured a mean flexural strength of 16.8 MPa [42]. The mean fracture toughness was 0.18 MPa m^0.5^, rather low, although the same test specification was referenced. The difference between the results of the present study and the reported values may have been influenced by the specimen geometry and slight variations between the methods, e.g., using encapsulated GICs or a different storage medium, and could also be influenced by the material composition of the GIC investigated in this study, i.e., the size of the filler particles or the molecular weight of the polyacrylic acid [43,44]. To ensure reproducibility of the results, ASTM standard E399 and ISO 9917-2 were used in the presented study for fracture toughness and flexural strength testing, respectively. For the use of ASTM standard E399, indicated for metallic materials, slight modifications had to be made to the specimen geometry, initial crack size, and loading rate in order to apply it to the materials tested. Although this does not affect the comparability of the results within this study, it makes it difficult to compare with other studies and is therefore a limitation of this study. The reinforced material investigated in this study could also show a significant increase in fracture toughness and resistance to cyclic loading compared to other conventional GICs [45]. In the mentioned study, class I cavities were prepared in extracted sound first molars and restored before the teeth were mechanically and thermally stressed using a chewing simulator with a maximum of 1,200,000 load cycles.

In the context of improving the mechanical properties of conventional GICs, it is essential to consider the mechanical properties of the natural tooth throughout the lifetime of individuals. There is evidence that the tooth structure becomes more susceptible to fracture with increasing age, indicating altered mechanical properties [46]. Whereas dentine is rather tough, tooth enamel shows generally a more brittle material behavior. In the case of enamel, mechanical properties also vary widely across the enamel layer due to the anisotropic arrangement of the enamel rods, thus resulting in a much greater crack resistance of the inner enamel [47]. Crack growth resistance values range from 0.11 ± 0.18 MPa m^0.5^/mm to 2.62 ± 1.39 MPa m^0.5^/mm for outer and inner enamel, respectively [48]. Given the heterogeneity of the mechanical properties of dentine and enamel, it is extremely difficult to define matching requirements for a homogeneous restorative material in order to cover the differences of natural tooth materials as best as possible. Considering this, improving the mechanical behavior of conventional GICs to increase their durability is the only valid option to expand clinical applicability.

Due to its chemistry, a typical conventional GIC shows a very brittle fracture behavior with almost pure elastic deformation before the material fractures. The reinforced GIC used in this study, on the other hand, has the potential to optimize the deformation characteristics due to the elastic nature of the dispersed PEG-PU micelles, thus increasing the toughness by ductile particle reinforcement of the material without compromising the maximum strength. A prerequisite for increasing the fracture toughness is sufficient coupling between the micelles and the surrounding matrix to be able to utilize the elasticity of the micelles in the toughening process. Microscopical investigations should address the toughening mechanisms within the glass ionomer material in future studies to support these theoretical considerations.

The mechanical properties of conventional GICs are closely related to their microstructure and influenced by the composition or ratio of glass and liquid, particle size of the glass fillers, and molecular weight of the polyacrylic acid [9,10,49]. This also includes the distribution and size of potential pores inside the material, which is particularly dependent on the mixing process of powder and liquid [50]. Automatically mixed encapsulated GICs tend to have a higher level of porosity in the final material than hand-mixed powder–liquid systems [51]. Using a hand-mixed system might therefore be beneficial to evaluate differences between materials to avoid an inhomogeneous microstructure with a higher level of porosity that might have influenced the results. However, manual mixing can instead slightly change the ratio between powder and liquid, which might lead to greater variations in the mechanical performance.

According to the ISO 10993-22 on the biological evaluation of medical devices, the novel GIC, investigated in this study, can be considered a nanomaterial as it contains dispersed micelles with dimensions between 1 nm and 100 nm [52]. In contrast to resin-modified GICs, however, where there is a risk of unpolymerized meth(acrylates) being washed out due to insufficient light activation, the dispersed micelles are firmly integrated in the cement matrix due to their hydrophilic and polar surface. Both PU and PEG are also common polymers used in the field of medical devices as part of drug delivery systems or in cardiovascular devices [53,54].

A limiting factor of the experimental setup of this study is that the optical verification of the dispersed PEG-PU nanoparticle was analyzed in an aqueous suspension and not in the final cured GIC, as the visibility of the incorporated micelles within the cured material would have been very limited due to their small size and superimposition by the much larger glass fillers. From the size and characteristics of the visible structures in the liquid suspension examined, it can be ruled out that the structures shown are aggregates of OsO_4_. Similar investigations with dendrimers and a comparable preparation method support the conclusion that the structures are in fact the dispersed micelles [55]. The objective of this study was limited to investigating the influence of the micelles on flexural strength and fracture toughness. Further investigations might also focus on other parameters that are crucial for bioactive direct restorative materials, such as fluoride release capability, wear resistance, and marginal integrity. Comparisons should also be made with other conventional GICs on the market, as well as with RMGICs and resin composites in order to better assess the potential of the material investigated in relation to alternative material concepts for similar clinical indications.

As a cause of considerable clinical shortcomings and a significant shorter longevity compared to resin composites [56], restorative materials based on conventional GICs are in general not indicated for use as a permanent solution for restorations subjected to high occlusal loads due to their limited mechanics. Mechanical improvements of direct restorative materials based on conventional GIC is therefore a mandatory prerequisite for clinical application in load-bearing areas. However, as increasing the mechanical properties of a conventional GIC in a laboratory setting is not yet sufficient to make reliable statements about the multi-factorial clinical performance of the restorative material [39,57], the indications of this novel restorative material are limited at this stage to be a permanent restorative solution for pediatric dentistry, class V cavities, and class I and II cavities that are not subjected to masticatory force. The next step should be to generate clinical evidence for a potentially permanent restorative solution in load-bearing areas to extend the indications accordingly. The relatively small sample size was selected due to the very controlled laboratory setup examining the flexural strength and fracture toughness of the investigated GICs. However, the results of the mechanical tests in this study should be interpreted with appropriate caution based on the size of the test groups. Future studies with a more clinical focus and a possible higher variability of the test conditions should be conducted with an increased sample number to enhance the clinical significance. In addition, measurements of the elastic modulus would be beneficial to draw conclusions about the deformation behavior of this novel material.

## 5. Conclusions

Based on the novel material concept presented in this study, the successful integration of the micelles into the liquid component of the GIC could be demonstrated and verified by TEM imaging. This study shows that reinforcing a conventional GIC with PEG-PU nanoparticles can significantly improve the flexural strength and fracture toughness and may expand the clinical applicability of this material class in direct restorative dentistry. Since the investigation of mechanical parameters in a laboratory setting only serves as a clinical predictor to a limited extent, clinical investigations should be carried out to confirm the high potential of this material concept. 

## Data Availability

The data that support the findings of this study are available from the corresponding author upon reasonable request.
